# Ozone potential to fight against SAR-COV-2 pandemic: facts and research needs

**DOI:** 10.1007/s11356-020-12036-9

**Published:** 2021-01-02

**Authors:** Angeles Blanco, Francisco de Borja Ojembarrena, Bernardino Clavo, Carlos Negro

**Affiliations:** 1 Chemical Engineering and Materials Department, Universidad Complutense de Madrid, Avda. Complutense s/n, 28040 Madrid, Spain; 2 Research Unit, Chronic Pain Unit, Dr. Negrín University Hospital, Calle Barranco de la Ballena, s/n, 35019 Las Palmas de Gran Canaria, Spain

**Keywords:** COVID-19, Coronavirus, Disinfections, Ozone gas, Heat & liquid sensitive materials, SARS-CoV-2

## Abstract

The greatest challenge the world is facing today is to win the battle against COVID-19 pandemic as soon as possible. Until a vaccine is available, personal protection, social distancing, and disinfection are the main tools against SARS-CoV-2. Although it is quite infectious, the SARS-CoV-2 virus itself is an enveloped virus that is relatively fragile because its protective fatty layer is sensitive to heat, ultraviolet radiation, and certain chemicals. However, heat and liquid treatments can damage some materials, and ultraviolet light is not efficient in shaded areas, so other disinfection alternatives are required to allow safe re-utilization of materials and spaces. As of this writing, evidences are still accumulating for the use of ozone gas as a disinfectant for sanitary materials and ambient disinfection in indoor areas. This paper reviews the most relevant results of virus disinfection by the application of gaseous ozone. The review covers disinfection treatments of both air and surfaces carried out in different volumes, which varies from small boxes and controlled chambers to larger rooms, as a base to develop future ozone protocols against COVID-19. Published papers have been critically analyzed to evaluate trends in the required ozone dosages, as a function of relative humidity (RH), contact time, and viral strains. The data have been classified depending on the disinfection objective and the volume and type of the experimental set-up. Based on these data, conservative dosages and times to inactivate the SARS-CoV-2 are estimated. In small chambers, 10–20 mg ozone/m^3^ over 10 to 50 min can be sufficient to significantly reduce the virus load of personal protection equipment. In large rooms, 30 to 50 mg ozone/m^3^ would be required for treatments of 20–30 min. Maximum antiviral activity of ozone is achieved at high humidity, while the same ozone concentrations under low RH could result inefficient. At these ozone levels, safety protocols must be strictly followed. These data can be used for reducing significantly the viral load although for assuring a safe disinfection, the effective dosages under different conditions need to be confirmed with experimental data.

## Introduction

COVID-19 is spreading in waves from one country to another country all over the world. Ten months after the outbreak in China, there are more than 54 million confirmed cases, growing at rates of up to 600,000 new cases/day, with an estimated fatality rate of 3% (Worldometer 2020). The greatest challenge today is to effectively control the extent of the pandemic spread until a successful vaccine is available.

SARS-CoV-2 belongs to the coronavirus family and causes illnesses ranging from those similar to that of the common cold to severe acute respiratory syndrome (SARS). It belongs to the Betacoronavirus genera in the family Coronaviridae of the order Nidovirales, and it is classified in Group IV according to the Baltimore classification. This group is characterized by having a positive single-stranded RNA. The genomic similarity to SARS-like coronaviruses obtained from bats is almost 90% and amino acid similarity to SARS 2003 and SARS-CoV-1 is almost 80%, which suggests that lessons learned from previous SARS outbreaks might be applied with caution until new data become available (Wu et al. 2020). As an enveloped virus, the virion, or the form the virus takes when outside the body, is wrapped in a fatty layer to protect it during transmission. If the envelope is damaged or dried out, the virus will die. Since this layer is sensitive to heat, detergents, solvents, and oxidants, the virus is relatively delicate to the external environment. This weakness has been used to control it (Swilling 2015).

The incubation period for humans is around 4 days (usually between 2 and 7 days). The most common symptoms of COVID-19 are fever and dry cough. Symptoms do not usually appear for a period ranging from 2 to 14 days or more, during which the asymptomatic patient is contagious (Guan et al. 2020). Indeed, it has been shown that between 5 and 80% of people with positive SARS-CoV-2 test may be totally asymptomatic, particularly so in children and young adults. This has been one of the main difficulties in controlling the pandemic (Oxford 2020).

Nowadays, it is recognized that potential mode of transmission of SARS-CoV-2 is aerosols and formites. The survival time of the SARS-CoV-2 virus depends on the environment. Existing evidence suggests that airborne transmission, particularly via nascent aerosols from human atomization, is very rapid and is a dominant route for the transmission of this disease, as was also the case in SARS-CoV-1 (Zhang et al. 2020). SARS-CoV-2 virus levels were as high as 67,164 copies/m^3^ in the air of hospital environments housing COVID-19 patients (Yao et al. 2020). Due to the possible build-up of the airborne virus-carrying droplets, the stability of the virus in indoor air is critical (Morawska and Cao 2020; Riddell et al. 2020). SARS-CoV-2 is stable for a period varying from several hours to several days in aerosols and on surfaces. The virus survives 3 h in aerosols, 4 h on copper, 24 h on porous surfaces such as cardboard, 48 h on stainless steel, and 72 h on plastic (Van Doremalen et al. 2020). These preliminary figures are, in general, lower than the 4–5 days estimated for the SARS-CoV-1 survival in metal, wood, and paper (Kampf et al. 2020). Recently, viable SARS-CoV-2 has been isolated after 7 days on surgical masks by Chin et al. (2020) and after 28 days from common non-porous surfaces (glass, stainless steel, banknotes) at ambient temperature and humidity by Riddell et al. (2020). Increasing the temperature drastically reduced the survivability of the virus to as little as 24 h at 40 °C.

So, besides the highly transmissible nature of COVID-19, these facts show it also has quite strong persistence. Because of this, it is now recognized that disinfection is more essential than ever. Until a vaccine is developed and/or a large portion of the population has been infected, to prevent SARS-CoV-2 virus spread, it is necessary to (1) remove the virus-laden droplets and aerosols from indoor air by ventilation or (2) inactivate or kill the virus, whether airborne or on surfaces, with adequate treatments.

Protocols for indoor environment disinfections of public places have been adapted and are continuously under review, but fast effective disinfection alternatives are still necessary for a safe resumption of usual business. Since the International Labour Organization estimates that 195 million jobs are at risk, best available disinfection protocols are being applied, in the first instance for health care facilities, nursing homes, hotels, and public transport, as well as for shops, offices, schools, restaurants, cruise ships, cinemas, etc., to minimize the socio-economic crisis. However, new outbreaks are arising after these practices have been adopted and, therefore, more efficient disinfection protocols are demanded to help to defeat the coronavirus.

The health crisis has been aggravated by the shortage of personnel protection equipment (PPE) and sanitary material. The lack of PPE represents a safety problem for health care workers and has resulted in a high number of them being infected. This situation is still happening in many regions, and it could recur in Europe if a larger wave returns. Accordingly, immediate solutions to extend the life of PPE and reuse them in a safe way have become essential to prepare against new shortages. Most of the small number of published works on disinfection of PPE and similar materials study the use of liquid chemicals, heat, or ultraviolet light (Ludwig-Begall et al. 2020), but all these methods present different drawbacks. For example, liquid treatments require a drying time, and residual products may produce skin irritation; some medical materials are heat or liquid sensitive; and ultraviolet light is not efficient in complex materials with areas in the shadow. Thus, the use of ozone gas has been proposed (Hudson et al. 2007; Lee et al. 2020) since it can easily penetrate to most areas and surfaces that require disinfection.

Ozone is a natural strong oxidant gas (2.07 V oxidation potential compared with 1.36 for chlorine), with a wide antimicrobial spectrum, very reactive to proteins and lipids, particularly with biological membranes. It has been widely used for water disinfection during the last century, and it is considered as one of the best biocides against microorganisms by the World Health Organization. Aqueous solutions of ozone are in use as disinfectants in many commercial situations, including wastewater treatment, laundries, and food processing, but the use of the gas itself is still under study. Ozone is very reactive and, as a consequence, is unstable. Residual ozone molecules break down naturally to oxygen with a half-life of 20–30 min at 30 °C (Hudson et al. 2009). Because of this, ozone must be produced and used in situ. It is easily generated from oxygen or air by ozone generators. Since 2013, it has been included in the EU Biocidal Products list and controlled by the 528/2012 Regulation. Thus, the active substance ozone and the ozone-generating equipment need to be authorized to be used in Europe (ECHA 2020). Ozone as active substance was authorized in 2016 while, according to article 93, in situ production of ozone for different uses is under review and subjected to national laws pending the final decision on its approval. In other countries like Japan, ozone was introduced in 2008, as part of the measures to prevent the swine flu infection, and it was authorized to be used in main airports (Nara 2020).

Fast disinfection alternatives are demanded not only for PPE but also for of all types of materials in contact with the patients as well as beds, rooms, and common areas. Therefore, the use of mobile ozone generators would facilitate its application in different areas.

On the other hand, the level of natural ozone in the environment also has a significant influence on the transmission of viruses. Ali et al. (2018) analyzed 20 years of influenza data in Hong Kong and demonstrated that when ambient ozone concentration level increased, the transmission ability of influenza viruses (H1N1, H3N2, and Influenza B) decreased substantially. With respect to COVID-19, Yao et al. (2020) have demonstrated that its transmission was negatively impacted by higher ambient average ozone level, higher temperature, and lower relative humidity. They found a statistically significant negative association between ozone levels (49–95 μg/m^3^) during Jan–March, 2020 and the confirmed number of COVID-19 cases. In general, a lower number of confirmed cases were observed when ozone level was higher than 73 μg/m^3^. Although the association of ambient O_3_ with reduced virus transmissibility may be related to the virucidal activity of O_3_ and its effect on host defense, this result implies that these environmental parameters might be adjusted to mitigate the transmission of COVID-19. If these data are confirmed, low concentrations of ozone (≈ 0.075 mg/m^3^) could be used indoors to mitigate the transmissions (e.g., in hospitals and nursing homes). This approach has been also suggested in a recent press release from Nara University.

Ozone has a significant disinfection potential against SARS-CoV-2, but it has to be used with caution following the protocols and regulations due to the risk of lung toxicity. At this moment, Nara Medical University has just confirmed the inactivation of the virus SARS-CoV-2 by exposure to ozone gas (Nara 2020), but the right doses and required concentrations to inactivate it in different environments, conditions, and surfaces are not known yet. Many companies already offer these treatments, despite the fact that there is still not enough scientific data on the safe dosages. A feeling of safety based on inefficient disinfection measures would represent an additional risk, since people could relax their personal protection.

Since research on COVID-19 is still developing, researchers are forced to apply knowledge about similar viruses. The aim of this paper is to review the most relevant results of virus disinfection by the application of gaseous ozone. The review covers treatments in different volumes, from small boxes and controlled chambers to larger rooms, as a base to develop future ozone protocols against COVID-19. Published papers have been analyzed to evaluate trends in the required ozone dosages, as a function of treated volume, relative humidity (RH), contact time, and viral strains. Based on these data, conservative dosages and times to inactivate the SARS-CoV-2 are estimated. These data can be used for reducing the viral load although for assuring a safe disinfection, the effective dosages under different conditions need to be confirmed with experimental data.

## Data analysis and discussion

Numerous studies have demonstrated that ozone can reduce the level of bacteria (Ishizaki et al. 1986; Kowalski et al. 2003; Sharma and Hudson 2008; Zoutman et al. 2011), of spores (Thill and Spaltenstein 2019) and of viruses (Kekez and Sattar 1997; Tseng and Li 2008; Tanaka et al. 2009; Hudson et al. 2009). As a gas, it can penetrate all areas within a room, including crevices, fixtures, fabrics, and the under surfaces of furniture, much more efficiently than liquid sprays, aerosols, or ultraviolet light. However, the ozone needs to be well distributed in large areas to minimize stagnant regions with lower ozone concentrations, and circulation fan maybe needed (Ito 2007). Ozone has been already used with the air conditioning system to sterilize bio-cleanrooms used for manufacture of sterile compounds (≥ 400 mg/m^3^; 80% RH; 2 h) using blow vents and suction ports to ensure a uniform airflow through the entire room during sterilization (Iwamura et al. 2012). Besides its antimicrobial activity by a direct action, ozone is also recognized for other indirectly mediated effects on biological systems, such as anti-hypoxic, analgesic, immune modulation, etc. These mechanisms of action have supported its use in different fields of medicine (Seidler et al. 2008; Swilling 2015), and ozone therapy is being studied against the cytokine storm induced by SARS-CoV-2, but this is outside the scope of this study.

Ozone acts on a broad range of targets in viruses, including the viral capsid, specific viral attachment epitopes into new cell hosts, and viral RNA (Torrey et al. 2019). It may inactivate viruses by both diffusing through the protein coat into the nucleic acid core, resulting in damage of the viral RNA, and by disrupting the exterior protein layer by oxidation. However, little attention has been given to elucidate the mechanism of virus inactivation by ozone, and the few published studies are focused on using aqueous solution of ozone. Katzenelson et al. (1978) reported a two-stage ozone inactivation curve of poliovirus type 1 when it was treated with ozonized water. They demonstrated a dose-response relationship between ozone and the inactivation ability. At a dosage of 1.24 mg/L, in the first fast stage, 99% of the viruses were inactivated in seconds, whereas the remainder of the viruses were killed during the second stage, which continues for several minutes. In 1981, Roy et al. associated this result with a change in the polypeptide structure of the protein coat although it did not cause disintegration of the viral particles. The reduced attachment could not account for the high percentage of inactivation obtained for a low residual concentration of ozone (0.3 mg/L with a contact time up to 2 min). Therefore, the major cause of inactivation was attributed in this case to RNA damage where the diffusion of ozone through the protein coat is the limiting step for ozonized water treatments. Ozone in water may act directly and through radicals while ozone gas acts only directly so the kinetics will be different. Mechanisms of virus inactivation by ozone gas are not available, but they would be useful in developing ozone disinfection processes.

Although there is a significant potential for ozone against SARS-CoV-2, it is a toxic compound, and risks and consequences of exposure of people to ozone gas has led to restrictions in its use. The efficiency of ozone depends on the total dosage, the concentration, the treatment time, the type of virus, and the environmental conditions (temperature and RH)  (Dennis et al. 2020). It is important to note that the concentrations required for disinfection are several magnitudes higher than the exposure limits defined for Occupational Safety and Health regulations (OSHA 2020). In general, the limits for workers are < 0.1 ppm or 0.2 mg/m^3^ as average in 8 h; < 0.3 ppm if exposure time is only 15 min; and if the concentration is > 0.3 ppm, a PPE is needed. In practice, this is overcome by ensuring that the area to be treated is closed to people and sealed. After treatment, which includes a decay time that varies depending on total dose and environmental condition, the area should be well ventilated. An improvement of this treatment could be achieved by combining ozone with UV irradiation which could result in the need of lower dosages and times. On the other hand, faster residual ozone removal can be achieved by using adsorbents and/or catalytic and heat converters.

According to the data from Yao et al. (2020), exposure to around 0.075 mg/m^3^ (0,038 ppm) in the presence of people might be useful to partially mitigate the COVID-19 transmission during this crisis. This level is below the limits for air ozone treatments in the presence of people. UNE 400-201-94 in Spain establishes that the maximum level for this case is 0.1 mg/m^3^ (0.05 ppm).

In recent decades, treatment of several viruses has been studied, representing different families and structural features. Data are available for DNA and RNA viruses with and without membranes. The papers show that all studied virus were susceptible to ozone, and, in most cases, they had similar inactivation kinetics on different hard surfaces (plastic, glass, and stainless steel) containing dry or wet films of the virus (Murray et al. 2008).

The ability of ozone to corrode certain materials after prolonged exposure has not been observed in the few data available on PPE disinfection. Lee et al. (2020) demonstrated the resistance of dust masks, such as N95, to ozone at concentrations of 120 ppm up to 5 min. These face masks are commonly composed of high-performance polymers and composites, such as non-woven polypropylene fiber, which allow its use under oxidizing atmospheres. Manning et al. (2020) proved that the exposure of N95 respirators, types 1860, 1870 and 8000, to ozone at 400 ppm for 2 h and 10 cycles did not show significant changes in filtration efficiency nor integrity and that it was safe for PPR reuse. Nevertheless, gaseous ozone is an extremely oxidizing agent, and severe effects can be observed when ozone is applied to devices made of natural rubber or derived composites (Lewis 2016). Composites of this natural polymer can be found in light, flexible, and resistant products, like high-performance non-flammable clothes (straps, boots), gloves, or some mattresses.

Since the experimental protocols are very diverse, the antiviral data of ozone have been grouped and analyzed considering two main groups: indoor environmental disinfection (aiming to treat the ambient air) and surface disinfection. In both cases, data are separated based on the experimental treated volumes.

### Environment viral disinfection

The experimental methodology of indoor air ozone treatment is common, and most of the authors use similar processes, regardless of the treated volume. The first step is the suspension of target viruses into water. After that, viral suspensions are aerosolized in the controlled chambers by using different devices including collision three-jet nebulizers (Tseng and Li 2006), collision six-jet nebulizers (Dubuis et al. 2020), and electric hot foggers (Pekovic and Kacimi 2015). The ozonation process takes place right after the aerosols are sprayed all over the indoor air environment. When the test is performed in small chambers, ozone is dosed through direct injection from a small tube because of the ease of diffusion all over the treated volume (Tseng and Li 2006; Dubuis et al. 2020). If the ozonated volume is a large room, the ozone generator is placed in the center of the room to allow access to the entire air volume, and it is dispersed by the use of fans (Pekovic and Kacimi 2015; Hudson et al. 2009). To confirm the effect of ozone disinfection, a control experiment is always performed by adding aerosolized viral suspensions to air without ozone treatment. Sampling process requires the use of impactors or samplers that collect microparticles of aerosols (around 1–1.2 μm of particle size) which obtain controlled air volume samples before and after the treatment (Tseng and Li 2006, Dubuis et al. 2020).

#### Small chambers (V < 55 L)

Several authors have studied the disinfection of small boxes and test chambers infected by spraying the viruses. Table 1 analyzes the data and compare infectivity with and without ozone in the different studies.

**Table 1:** Efficiency of ozone against viruses at several conditions in small chambers. Virus of Group IV are italicized

Virus	RH (%)	O_3_ (mg/m^3^)	Time (min)	TC (mg/m^3^·min)	Viral reduction	Ref.
*Levivirus MS2*	55	4.5	0.31	1.38	99%	Tseng and Li (2006)
		12	0.23	2.76		
		2.26	40	90.4		Dubuis et al. (2020)
	85	5.6	0.31	1.72	99%	Tseng and Li (2006)
		8	0.23	1.84		Dubuis et al. (2020)
		2.26	10	22.6		
Sinsheimervirus ϕX174	55	3.6	0.31	1.10	99%	Tseng and Li (2006)
		7	0.23	1.61		Dubuis et al. (2020)
		2.26	40	90.4		
	85	3.2	0.31	0.98	99%	Tseng and Li (2006)
		5.2	0.23	1196		
		2.26	10	22.6		Dubuis et al. (2020)
Cystovirus ϕ6	20	2.26	10	22.6	99%	Dubuis et al. (2020)
	55	2.8	0.31	0.86	99%	Tseng and Li (2006)
		5.2	0.23	1196		
	85	2.5	0.31	0.77	99%	Tseng and Li (2006)
		4	0.23	0.92		
		2.26	40	90.4		Dubuis et al. (2020)
Teseptimavirus T7	55	11	0.31	3.37	99%	Tseng and Li (2006)
		19	0.23	4.37		
	85	7	0.31	2.15	99%	
		15	0.23	3.45		
Tectiviridae PR772	20	2.26	10	22.6	99%	Dubuis et al. (2020)
	85	2.26	10	22.6		
*Murine norovirus*	85	0.46	40	18.4	99%	Dubuis et al. (2020)
Sendai virus (HVJs)	50	400	30	12,000	96.02%	Sato et al. (1990)
	60	400	30	12,000	98.00%	
	70	400	30	12,000	99.94%	
	80	400	30	12,000	99.97%	
	80	400	60	24,000	99.99%	
	90	400	30	12,000	99.98%	
Theiler’s murine encephalomyelitis virus	80	400	180	72,000	99.99%	Sato et al. (1990)
Murine hepatitis virus	80	400	30	12,000	99%	Sato et al. (1990)
	80	400	120	48,000	> 99.99%	
	80	600	30	18,000	99%	
	80	600	120	72,000	> 99.99%	

The influence of RH is very important, and in many cases, if ozone use is considered, it will determine the best disinfection doses. This fact is in agreement with the data recently published by Guillier et al. (2020) on coronavirus inactivation.

**Fig. 1:**
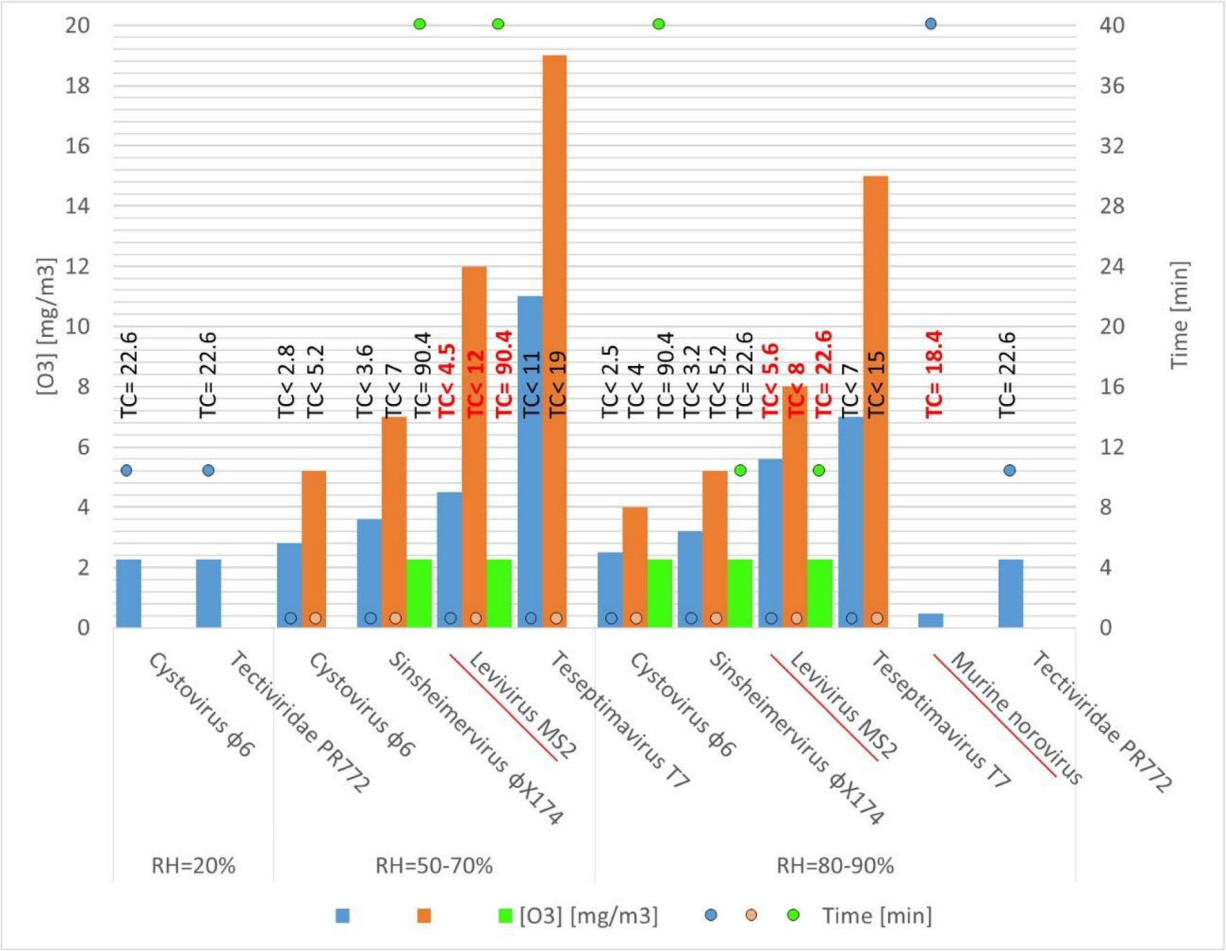
Ozone concentrations and contact times required for 99% viral inactivation in low-volume spaces. Virus of Group IV is underlined and marked in red

The levels, time, and RH conditions required for a 99% (2-log) for different viral strains are summarized in Fig. 1.

In most cases, ozone total concentrations (TC = level (mg/m^3^) × treatment time (min)) of no more than 100 mg/m^3^ min are required for a 99% reduction of the virus load. Levels from 0.5 to 20 mg/m^3^ and times from < 1 to 40 min have been studied. High ozone concentrations are effective at short time of exposure (< 1 min) regardless of the virus. When ozone concentration is below 5 mg/m^3^, longer periods of time was needed to reduce viral concentration, in some cases as high as 40 min. At higher RH, the levels required are up to 40% lower.

These estimated data are in agreement with the very recent experimental results from Nara Medical University about PPE disinfection which obtain up to 99% virus decrease with a TC of 120 mg/m^3^ min and up to 99.99% virus decrease with 660 mg/m^3^ min, which is the TC value required for the certification of medical devices by the Ministry of Health, Labour and Wellness in Japan (Nara 2020).

Sato et al. (1990) achieved higher viral reductions (up to 99.99%), but the ozone concentration was higher compared with the other studies (400 mg/m^3^, 1 or 3 h at 80% RH thus TC ≥ 24,000 mg/m^3^ min were required, depending on the virus).

Previous studies show that norovirus has a high stability and resistance toward environmental stress and is much more resistant than coronavirus toward alcohols, chlorine, and ultraviolet disinfection (Li et al. 2020). On the other hand, Dubuis et al. have treated airborne norovirus with ozone, in a controlled chamber, under high RH conditions, low ozone concentration (0.46 mg/m^3^ or 0.23 ppm), and 40 min of contact time (Dubuis et al. 2020). The reduction of murine norovirus infectivity was as high as 99.8%. This fact shows that a large reduction of infectivity can be achieved treating the atmosphere with aerosolized norovirus even when the concentration of ozone is as low as 0.46 mg/m^3^ when the RH is as high as 85%. Therefore, considering the similitude between SARS-CoV-2 and murine norovirus, one might expect, from Fig. 1, that TC doses of 20 mg/m^3^ min could be efficient for SARS-CoV-2 under high RH conditions (this has to be confirmed by experimental studies).

Lee et al. have shown, using a human coronavirus (HCoV-229E) as a surrogate for SARS-CoV-2, that the virus present on contaminated masks lost its infectivity to a human cell line (MRC-5) when exposed to 240 mg/m^3^ ozone gas during 1 min. Similar results were obtained for influenza A virus (H1N1). In this case, this short exposure time did not fully degrade the viral RNA, and thus, the loss of infectivity was attributed to the damage of the viral envelope or envelope proteins, resulting in failure of the virus to attach itself to host cells (Lee et al. 2020).

In summary, from a conservative point of view, TC of 100–200 mg/m^3^ min are expected to assure a high inactivation of the virus, and they could be easily applied to decrease the viral load of PPE and other materials in small chambers during pandemic peaks, using small available ozone generators (2–3 g/h), at levels of 10–20 mg/m^3^ (5–10 ppm) for 10 min.

#### Larger spaces (V = 2–65 m^3^)

**Fig. 2:**
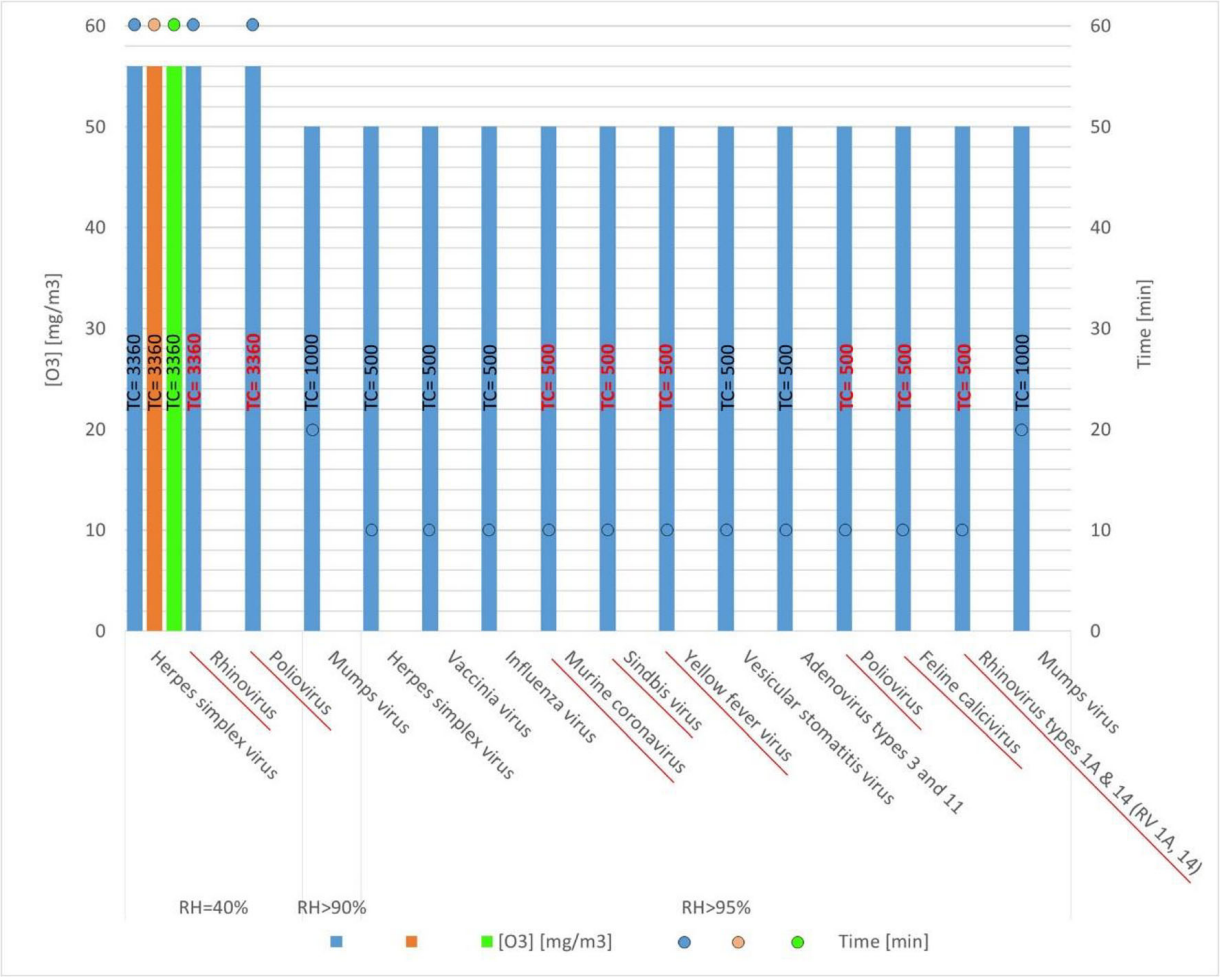
Ozone concentrations and contact time in different viral inactivation experiments of indoor air of wide spaces. RH = 40% **→** viral inactivation of: 95–99; RH ≥ 90% **→** 99.99% to 8-log reduction. Virus of Group IV is underlined and marked in red

Table 2 and Fig. 2 summarize the dosages obtained for viral inactivation in a large range from 95 to > 99.99%. All analyzed studies were carried out at different conditions in wide spaces like rooms, offices, laboratories, containing normal furniture.

**Table 2:** Efficiency of ozone against viruses at several conditions in large chambers. Virus of Group IV are italicized

Virus	RH (%)	O_3_ (mg/m^3^)	Time (min)	TC (mg/m^3^ min)	Viral reduction	Ref
Herpes simplex virus	40	56	60	3360	97.89%	Hudson et al. (2009)
	95	50	10	500	> 99.9%	Hudson et al. (2009)
*Rhinovirus*	40	56	60	3360	99%	Hudson et al. (2009)
	95	50	10	500	> 99.9%	Hudson et al. (2009)
*Poliovirus*	40	56	60	3360	> 99%	Hudson et al. (2009)
	95	50	10	500	> 99.9%	Hudson et al. (2009)
Vaccinia virus	95	50	10	500	> 99.9%	Hudson et al. (2009)
Influenza virus	95	50	10	500	> 99.9%	Hudson et al. (2009)
*Murine coronavirus*	95	50	10	500	> 99.9%	Hudson et al. (2009)
*Sindbis virus*	95	50	10	500	> 99.9%	Hudson et al. (2009)
*Yellow fever virus*	95	50	10	500	> 99.9%	Hudson et al. (2009)
Vesicular stomatitis virus	95	50	10	500	> 99.9%	Hudson et al. (2009)
Adenovirus types 3 and 11	95	50	10	500	> 99.9%	Hudson et al. (2009)
*Feline calicivirus*	95	50	10	500	> 99.9%	Hudson et al. (2009)
Sinsheimervirus ΦX174	70	0.152	36	5.472	99.99%	de Mik and de Groot (1977)
	74	0.142	30	4.26	97.60%	de Mik et al. (1977)
Mumps virus	> 90	50	20	1000	8-log	Pekovic and Kacimi (2015)

These data show again the influence of RH on indoor air ozone disinfection. All experiments carried out at high RH reached more than 99.9% reduction of the viral load. TC of 400–500 mg/m^3^ min were efficient to reduce the virus loads of a large number of viruses. Most studies were carried out at 50 mg/m^3^ (25 ppm) over 10 min (lower doses were not studied). Several viruses are in the same group as SARS-CoV-2 (Group IV): rhinovirus, poliovirus, murine coronavirus, sindbis virus, yellow fever virus, and feline calcivirus. The required contact time clearly depends on the RH. High contact time of 60 min is required when RH is 40% (typical value for a room or office is 40–60%) while at high RH (> 90%), 10–20 min is enough. This fact means that at low RH, 5 times higher contact times are required under similar ozone concentrations.

In any further disinfection studies, it will be important to carefully detail the operating conditions to allow the comparison of the results and recognize any practical implications. Similar concentration and time of ozone exposure could have different disinfection effects in different cities according to their ambient conditions (RH and temperature). Prior to the availability of any more comprehensive data, conservative high doses should be used to minimize the risk of a false feeling of safety.

### Surface disinfection of virus

The experimental set-up and methodology used for surface disinfection can be divided in different strategies. Ozonation of large spaces can be performed as said in 2.1 due to the fact that the aerosolization of extensive surfaces like walls, doors, or ceilings ensures the proper distribution of the viruses all over the studied surface, and the small size of drops and particles increases the contact area between ozone and the viral suspension on the surface (Pekovic and Kacimi 2015). Other option is to prepare sterile strips of different materials (steel, glass, plastic) with viral suspensions films spread over the surface. These strips must be manipulated in sterile cabinets to avoid contamination. Once dried, they can be transported in sterile containers to the test room, where they are placed in selected places of the room (walls, beds, doors) to perform the ozonation treatment to disinfect. In this case, two control experiments are needed: one including some strips that stay at the safety cabinet during the entire experiment and another one which consists of some strips contained within sterile sealed boxes that are placed in the test room but are not exposed to ozone treatment (Hudson et al. 2009).

In the case of ozone treatment of small volume chambers, there are some similarities in the methodology used in the different studies (Predmore et al. 2015; Brié et al. 2018; Tanaka et al. 2009; Lin et al. 2007; Cannon et al. 2012; Maier and Chu 2016; Zhou et al. 2018). The analyzed surfaces vary from inert materials like plastic (Tanaka et al. 2009) or stainless steel (Maier and Chu 2016) to microbiologically active surfaces like vegetables and fruit (Predmore et al. 2015; Brié et al. 2018; Zhou et al. 2018) or culture dishes (Lin et al. 2007). The most common steps in the ozone treatment are the following ones: firstly, the recovery of the lyophilized viral strains and the preparation of the viral suspension are necessary. Then, the viruses are applied to infect well-known bacterial strains, commonly guided and selected by a standard method. Once bacteria were inoculated, in all the cases, the dispersion methodology was spotting or adding drop-wise until covering the entire extension of the ozonized area. In some cases, surface drying step before ozonation is also mentioned (Tanaka et al. 2009; Zhou et al. 2018). Once finished, viruses are recovered through a specific treatment which depends on the surface and varies from sterilized water (Tanaka et al. 2009) to different extractives like phosphate-buffered solution (PBS) (Predmore et al. 2015; Lin et al. 2007), vegetable buffer (Zhou et al. 2018), or TGBE buffer (Brié et al. 2018). Viral quantification is performed by standard plaque assay method (Zhou et al. 2018) or viral titration, which means a quantitative assay of the infectivity of the virus recovered in monolayer cells of selected bacterial strains (Predmore et al. 2015; Lin et al. 2007; Brié et al. 2018; Tanaka et al. 2009). Other typical quantification methods mentioned by some researchers include reverse transcription polymerase chain reaction (RT-PCR), analysis of viral proteins, virus extraction (Brié et al. 2018; Predmore et al. 2015), and cytokine measurement (Lin et al. 2007) quantification methods.

There are a large number of factors influencing surface disinfection, including the type material. In general terms, high viral inactivation is attained when applying ozone disinfection treatments to inert materials (steel, glass, plastic, walls) achieving more than 3 log reductions (99.9%) in almost all the reported studies. On the other hand, biologically active materials, like vegetables or wood, show higher survival rates of virus (90–99% of viral reduction). This might be due to the presence of biological antioxidants.

It is noteworthy that only a small number of studies report RH conditions. While being one of the most important parameters when disinfecting airborne viruses, RH is commonly not shown or measured when disinfecting surfaces.

#### Small chambers (V < 55 L)

**Fig. 3:**
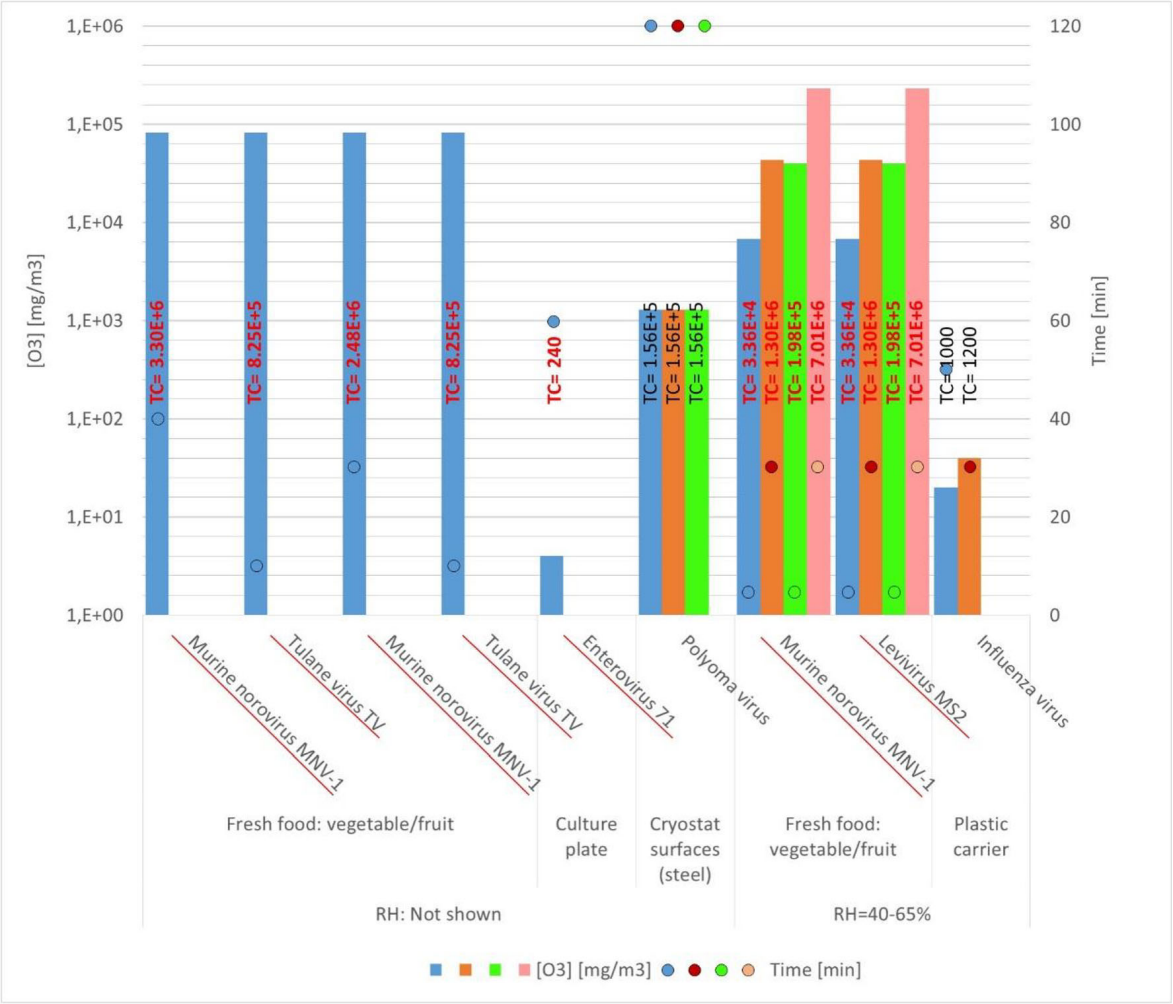
Ozone concentrations and contact time in different viral reduction experiments of surfaces in low-volume spaces. Viruses of Group IV are underlined and marked in red

Table 3 and Fig. 3 summarize ozone concentration, contact time, RH, and viral strains from published studies using low-volume spaces like boxes and test chambers.

**Table 3:** Efficiency of ozone against viruses on surfaces at several conditions in small chambers. Virus of Group IV are italicized

Virus	RH (%)	Surface	O_3_ (mg/m^3^)	Time (min)	TC (mg/m^3·^min)	Viral reduction	Ref.
*Murine virus*	40–50	Fresh food: fruit/vegetables	6729	5	33,647	92.06%	Zhou et al. (2018)
			43,415	30	1,302,450	97.49%	
			39,580	5	197,900	92.06%	
			233,502	30	7,005,060	98.41%	
	52		6	1	6	99.95%	Brié et al. (2018)
	No data		82,501	40	3,300,040	99.99%	Predmore et al. (2015)
			82,502	30	2,475,030	99.84%	
*Tulane virus TV*	No data	Fresh food: fruit/vegetables	82,501	10	825,010	99%	Predmore et al. (2015)
			82,501	10	825,010	99.68%	
Influenza virus	65	Plastic carriers	20	No data	900–1000	99.99%	Tanaka et al. (2009)
			40	No data	1200		
*Enterovirus 71*	No data	Culture plate	4	60	240	99.9–99.99%	Lin et al. (2007)
Polyoma virus	No data	Cryostat surfaces: steel	1300	120	156,000	99.999%	Maier and Chu (2016)
			1300	120	156,000	99.92%	
			1300	120	156,000	99.9995%	
*Levivirus MS2*	40–50	Fresh food: fruit/vegetables	6729	5	33,647	98.41%	Zhou et al. (2018)
			43,415	30	1,302,450	99.21%	
			39,580	5	197,900	99.37%	
			233,502	30	7,005,060	99.95%

Most of these studies are focused on achieving the maximum viral reduction, and therefore, the studied ozone dosages are extremely high, especially for vegetable disinfection. Plastic needs 20–30 mg/m^3^ (10–15 ppm) and 30–50 min of treatment to achieve 99.99% reduction of influenza virus. Stainless steel surfaces of a cryostat treated for 120 min with 1.300 mg/m^3^ of ozone achieved 5-log reductions of polyoma virus. Presumably, lower dosages and times could have been enough for a 99% reduction of virus load. Higher RH (> 90%) enhances the disinfection process, reducing the required ozone concentration and contact time. The best results of disinfection on inert materials are found when a controlled chamber is used, such as a culture dish and plastic carriers. The disinfection of a complete cryostat is difficult because this device has a large number of parts, and the ozone gets dispersed all over the equipment. As a result, it is difficult to achieve high concentrations of ozone in each part of the device, and the complete process needs 120 min.

Recent studies from Blanchard et al. (2020) confirm the influence of RH on virus disinfection efficiency with ozone. When using 40 mg/m^3^ of ozone, RH higher than 50% is needed to reach significant reduction of the Influenza Virus A (99.99%) in face masks, and 80% of RH is needed to achieve the same yield in N95 and Tyvek. These disinfection processes were carried out during 90 min, in the case of Tyvek and face masks, and 18 min in the case of N95, meaning a total CT of 3.600 mg/m^3^ min for face masks (RH = 62%) and Tyvek (RH = 80%) materials and a CT of 720 mg/m^3^ min for N95 masks. Compared with the results of Tanaka et al. (2009) under similar RH conditions (60%), facemasks required a CT value three-times higher to reach similar reduction values. The main reason is that Blanchard et al. (2020) did not study times shorter than 90 min at 62% RH, but according to Tanaka, this time can be shorter. Recently, Clavo et al. (2020) in a study focussed on the total removal of the SARS-COV-2 have demmonstrated that the effect of ozone was highly dependent on the RH. Under 99% RH a dosage of 8-13 mg/m^3^ during 30 min completely remove the virus from gown samples and highly reduced gene amplification in face masks. However, at 66% RH, the virus gene amplification was still detected after an exposure at 6-24 mg/m^3^ during 50 min, although it was an important decrease in gene amplification.

#### Wide spaces (V = 2–65 m^3^)

**Fig. 4:**
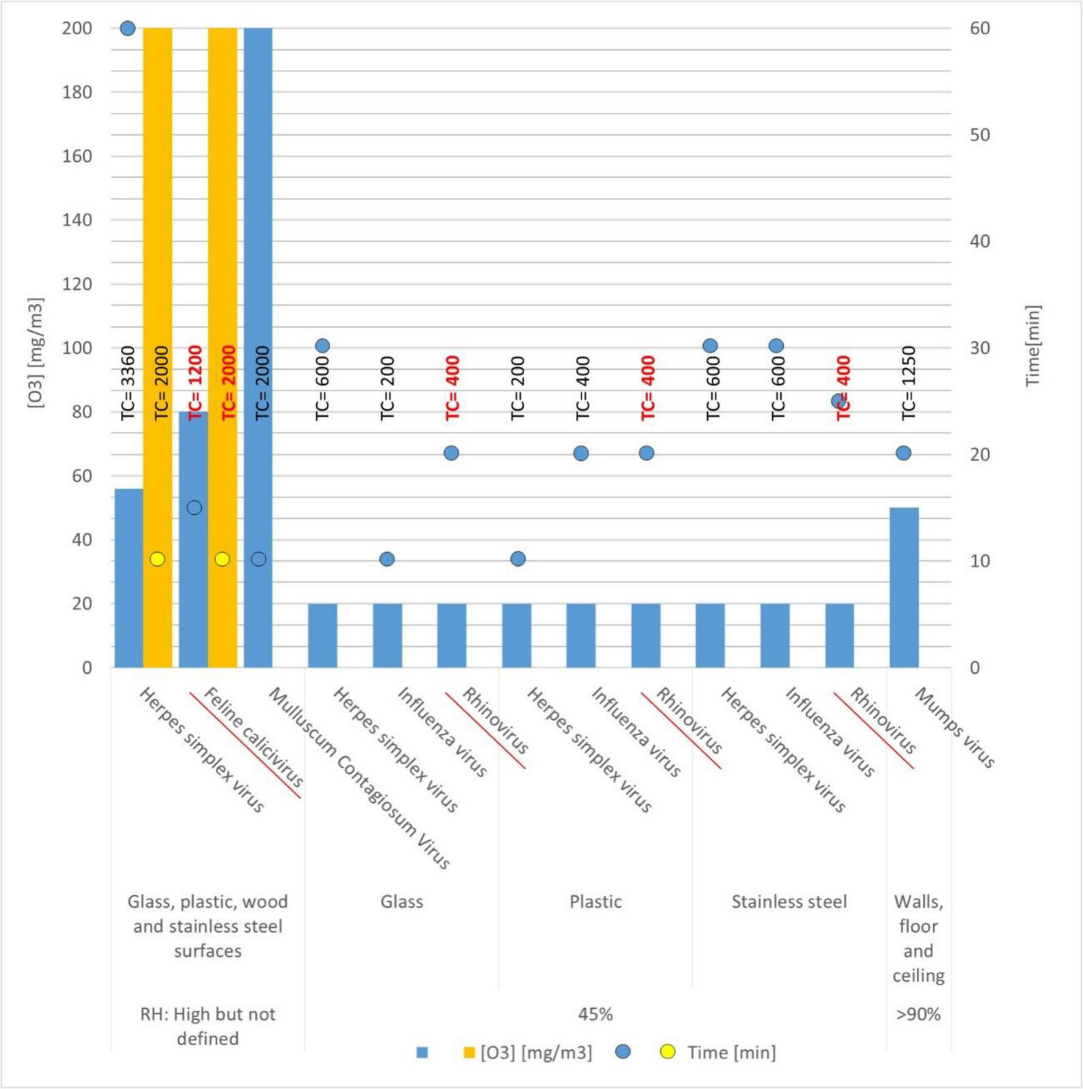
Ozone concentrations and contact time in viral inactivation experiments on surfaces in wide spaces. Group IV viruses are underlined and marked in red

Table 4 and Fig. 4 show the surface inactivation data for ozone treatment in rooms under different conditions (Boast et al. 2008; Hudson et al. 2009; Pekovic and Kacimi 2015). Viral inactivation varies from 95% to ≥ 99.99. All high RH experiments reached an inactivation higher than 99.9%.

**Table 4:** Efficiency of ozone against viruses on surfaces at several conditions in large chambers. Virus of Group IV is italicized

Virus	RH (%)	Surface	O_3_ (mg/m^3^)	Time (min)	TC (mg/m^3·^min)	Viral reduction	Ref.
Herpes simplex virus	45	Glass	20	30	600	100%	Hudson et al. (2009)
		Plastic	20	10	200		Boast et al. (2008)
		Stainless steel	20	30	600		
	High	Glass, plastic, wood and stainless steel surfaces	56	60	3360	90–95%	
			200	10	2000		
		Varied surfaces	56	60	3360	97.8–99.4%	
Influenza virus	45	Glass	20	10	200	100%	Hudson et al. (2009)
		Plastic	20	20	400		
		Stainless steel	20	30	600		
*Rhinovirus*	45	Glass	20	20	400	90%	Hudson et al. (2009)
		Plastic	20	20	400		
		Stainless steel	20	20	400	80%	
*Feline calicivirus*	High	Glass, plastic, wood and stainless steel surfaces	80	15	1200	90–95%	Boast et al. (2008)
			200	10	2000		
		Varied surfaces	80	15	1200	99.91%	
Mulluscum Contagiosum virus	High	Glass, plastic, wood and stainless steel surfaces	200	10	2000	90–95%	Boast et al. (2008)
Mumps virus	> 90%	Floor	50	25	1250	99.9997%	Pekovic and Kacimi (2015)
	> 90%	Ceilings	50	25	1250	99.9998%	
	> 90%	Walls	50	25	1250	99.9997%

Most of the experiments are carried out using concentrations closer to those used when air disinfection is also desirable. High ozone concentration experiments are carried out to achieve the maximum reduction in short exposure time. The time required is lower than in the rest of the studies, but the concentration is ten times higher. A conservative approach could be to apply of 20 to 50 mg/m^3^ (10–25 ppm) of ozone during 20 to 30 min to achieve a high virus reduction on surfaces in short times. Thus, a TC of 400–1500 mg/m^3^ min seems to be required which is lower than the 2400 mg/m^3^ min determined by Tanaka et al. (2009) for a 99.9 reduction of the influenza virus. Low concentrations for a longer time could be applied when time is not a limiting factor, as in the case of ozonization treatments at night in public facilities such as health centers, nursery common rooms, schools, and this has already been suggested by Tanaka et al. (2009).

## Conclusions

Antiviral and antimicrobial properties of ozone are well documented, and the potential for ozone gas against COVID-19, to decontaminate rooms and specific materials, is high. It is important to remark that safe treatments need to be carried out following adequate protocols by trained people.

The state of the art in treating other viruses suggests that conservative doses of 10–20 mg/m^3^ (5–10 ppm) for times varying between 10 and 50 min would be enough for disinfection of PPE and materials in small chambers. This corresponds to a TC of 100 up to 1000 mg/m^3^ min depending on the desired viral reduction. Long contact times (30–50 min) are required when > 99.99% of virus reduction is needed (TC = 900 mg/m^3^ min), but lower dosages are enough for a significant viral load reduction (TC = 200 mg/m^3^ min). In large rooms, 30 to 50 mg/m^3^ of ozone would be required for 20–30 min. Higher doses can be used for fast treatments. If time is not critical, lower doses of 5–10 mg/m^3^ and long times up to 4 h could be used to reached 1000 mg/m^3^ min. In all cases, maximum antiviral efficacy is achieved at high humidity (> 90%). In different areas, optimal doses will be very different, and at low RH, up to 5 times higher TC may be necessary for some applications.

Very low doses (< 0.1 mg/m^3^) in the presence of people could be useful to partially mitigate the COVID-19 transmission during this crisis.

These data will help researchers define their experimental variables and thus reduce their research time, and they will guide companies in the development of disinfection protocols.

Ozone gas disinfection is not officially recommended because in situ production and application is still being studied by ECHA, and its efficiency against SARS-CoV-2 has not been well proven yet. Specific data on effective doses for different conditions, according to each application, are needed to develop safe disinfection protocols. Specific studies with masks, respirator materials, and different materials in contact with COVID-19 patients are needed. Based on new data, the conservative doses discussed in this paper could probably be optimized and reduced. Studies are ongoing.

## Data Availability

The datasets developed during the current study are available from the corresponding author on reasonable request.
